# Quality of Life and GAF in Schizophrenia Correlation Between Quality of Life and Global Functioning in Schizophrenia

**Published:** 2011

**Authors:** Seyed-Hamzeh Hosseini, Mahtab Karkhaneh Yousefi

**Affiliations:** Psychiatry and Behavioral Sciences Research Center and Department of Psychiatry, Mazandaran University of Medical Sciences, Sari, Iran.

**Keywords:** Global function, Quality of life, Schizophernia

## Abstract

**Objective:** Recently, quality of life is a concern of health in psychiatry. Schizophrenia is a disorder that has the most regressive effects in societies’ and patients’ behavioural, occupational and psychiatric aspects. The main purpose of this study was to evaluate the quality of life and global function of schizophrenia patients.

**Methods:** A hundred schizophrenia patients (with DSM-IV-TR) who had a history of at least 10 years from the beginning of the disorder were collected. Demographic characteristics, type of schizophrenia, living condition and the quality of life scale (QLS) including: interpersonal relationship, instrumental role, intra psychic foundations and common objects and activities, were gathered. Patients' general functions were evaluated with Global Assessment of Functioning (GAF). Statistical analysis was performed by SPSS statistical software using Fisher’s exact test, analysis of variance (ANOVA), Dunken and Pearson’s correlation test.

**Results:** In this study 67% of patients were male and 51% were female. They were comprised of residual (55%), paranoid (11%) and undifferentiated (33%) schizophrenia. They lived in institute (67%) and with their families (28%). There was a moderate correlation between QLS and GAF (p<0.01, r=0.2). A significant association was found between married and single patients in instrumental role (F: 2.97), P<0.05) but there was not a significant association in other domains. Correlation were found between undifferentiated and paranoid patients in interpersonal relationship (F: 2.97), P<0.05). However there weren’t any correlation in intra psychic foundation and common objects and activities domains. Also there were significant associations in GAF (F: 3.98), P<0.05) between uneducated and educated participants. There was not an association between the Mean of five domains and genders.

**Conclusion:** Schizophrenia patients' quality of life is low which shows the value and reliability of Global Assessment Functioning that is usually used for every psychiatrics’ patients for V axis clinical diagnosis and indicated the clinical value of this scale. For this reason rehabilitation, social skill training of patients and supportive therapy in family are important.

## Introduction

Schizophrenia is a clinical syndrome of variable, but profoundly disruptive, psychology that involve cognition, emotion, perception, and other aspects of behavior. The expiration of these manifestations varies across patients and over time, but the effect of the illness is always severe and is usually long lasting. Schizophrenia is a global health disorder which costs a huge amount of personal and economical expense. Whereas it starts from the first years of life, it causes a lot of sever, long lasting impairment, and requires clinical admission, rehabilitation and supportive services (1,2).

Recently, improvement of the quality of life has become an established component of psychiatric treatment, although it will be hard to perform in a severe disorder such as schizophrenia (3). During last fifteen years, the huge amount of researches about patients with severe psychological disorders show that, personal and social variables could be effective on quality of life and recognizing them is necessary for quality of life promotion (4).

Recent research indicated that there is a relation between some clinical variables such as depression (5), anxiety (6), psychological pressure (7), positive symptoms (8), negative symptoms (9) and personality (10) in schizophrenic patients.

A number of cross-sectional studies show the relationship between some social and clinical variables and quality of life (11). Quality of life has different subjective and objective domains. Subjective domains include aspects such as well being, life satisfaction and happiness. The objective domains include social and environmental settings. Recent data suggest that there is severe functional impairment across a broad range of domains in Schizophrenia (12).

In addition, several long-term studies have shown that about 25 percent of patients can be described as having a good outcome. About 50 percent showed partially relieved and 25 percent had poor outcome (13).

The aim of this study was to evaluate the correlation between quality of life and global functioning in schizophrenic patients whom at least had more than 10 years passed from their onset of disease and to compare the results of this study and western countries’ studies about the effective factors on quality of life.

Whereas the culture and developing factors of countries have certain effect on clinical prognosis and outcomes of the disease (14, 15, 16, 17), we aimed to perform this study in Mazandaran province (north of Iran). We did not find any similar study by the searching in some databases such as Iranmedex with quality of life, Schizophrenia and global assessment keywords. Also there are limited studies in our country about this topic which none of them evaluated the correlation between quality of life and global assessment (the aim of this study). 

## Materials and Methods

First of all, we collected the name, address and telephone number of all patients diagnosed with schizophrenia who were hospitalized in Zareh Psychiatric Hospital of Mazandaran in 1999-2000 and their onset of disease were since 1996 and before. 

Then 100 Patients were randomly selected (using randomization table) among the 300 patients. Patients families were invited to a patient assessment interview in Zareh Psychiatric Hospital after the objectives of the study had been explained to them by telephone. If any patients diagnosed with schizophrenia refused to participate in the study, another patient would have been randomly chosen and enrolled to the study. 

Ethical approval was obtained and the written informed consent was obtained from the patients and families who participated in the study. The patients and their families were psychologically assessed by a senior psychology resident and answered to the researcher’s questions regarding demographic chart, quality of life scale and Global Assessment of Functioning GAF. The demographic chart had a number of variables such as age, gender, marital state, educational level, and living area. 

The quality of life scale QLS consists of 21 items in four domains: interpersonal relations (8 questions), instrumental role (4 questions), intra psychic foundations (7 questions), and common objects and activities (2 questions). In this questionnaire the range of score for interpersonal relations is 0 to 48, for instrumental role is 0 to 24, are intrapsychic foundations 0 to 42, and is common objects and activities 0-12. The total quality of life had scored from 0 to 126, which the higher scores reflect better quality of life. Every question has 6 options and scored from 0 to 6. The total score in domains is the average of scores in each domain. 

For the schizophrenic patients, the QLS questionnaire demonstrated acceptable content and construct validity and high reliability (Cronbach’s Alpha: 84-97) (18, 19). 

The instrument QLS was translated to Persian and was revised by two psychiatrist and it was used in Iranian population before. (20). 

GAF is a global functioning assessment in V axis of diagnosis of psychiatric patients which is usually used for psychiatrics’ outpatients and inpatients in three domains; social functioning, personal functioning, and psychological functioning. This scale was scored 0 to 100, which the higher scores reflects higher function in every domain. Statistical analysis was performed by SPSS using fisher’s exact test, Dunken and Pearson’s correlation test. 

## Results

We enrolled 100 outpatients in the study, which have chronic schizophrenia more than 10 years and have been at least one time admitted to Zareh Psychiatry Hospital and were diagnosed with DSM-IV schizophrenia in their history. 

1- Sixty nine percent of patients were men and 31% were women. Mean age of patients was 39 (21-60) lower-upper. Demographic characteristics of the patients are summarized in table 1. Data regarding characteristics of disease is summarized in table 2. 

As shown in table 1, the mean scores of impulsivity and its components were higher in suicide-ideators compared with non suicide-ideators.

**Table 1:** Demographic variables of patients with schizophrenia

Number/percent †		
Gender	Men Women	69 31
Age(year)	21-30 31-40 41-50 <50	12 40 35 11
Marital state	Single Married Windowed	42 39 19
Education	No educated Primary school High school University	14 39 31 9
Occupation	Unemployed Employed	98 2
Birth day	Spring Summer Autumn Winter	28 45 12 15
Smoking	Smoke Nonsmoking	53 47

†Considering the number of enrolled patients the digit shows both the number and percent

2- Quality of life, global functioning 

The scores of different quality of life domains including: interpersonal relations, instrumental role, intrapsychic foundations, and common objects and activities and total quality of life score and also the mean of GAF are showed in table 3. 

**Table 2:** Illness characteristics of the studied patients

Number/percent†		
Schizophrenia type		
	Paranoid	11
	Undifferentiated	33
	Residual	55
Age at onset (years)		
	< 20	48
	>20	52
Number of hospitalization		
	Once	30
	2-4 times	37
	>4	33
History of psychiatric		
disorder in first	Have had	32
degree relatives	Did not	68

†Considering the number of enrolled patients the digit shows both the number and percent

**Table 3:** The quality of life scores (QLS) and global assessment of functioning (GAF) scores

Effect	F	Hypothesis df	Error df	Sig.	Partial Eta Squared	Observed Power
Group	3.83	3	336	0.001	0.30	0.81

3 -The relationship between Quality of life domains and GAF by the demographic characteristics 

3-1: Comparison between mean of 5 QLS domains or GAF among gender, marital status, educational level and schizophrenic type with analysis of variance ANOVA revealed that: 

The total quality of life score among men is higher than women (p=0.039). Also there is a relation between interpersonal relations domains and gender. It means the men showed better interpersonal relations (p= 0.04). There is no significant difference among remaining domains regarding GAF. 

*3-2:* Concerning marital status, although the total quality of life score is higher in married patients than single ones but the difference was not significant. Also there is a significant difference among interpersonal relations domains and gender. Men showed better interpersonal relations (p= 0.04). Remaining domains and Global Functioning did not differ significantly among genders. 

3-3: years of education and GAF mean analyzed with ANOVA: The result indicated significant differences GAF mean among no educated (no educated or primary levels) and educated (high school or university) patients (p<0.04). But the quality of life domains did not differ significantly among these groups. 

3-4: Schizophrenia type and GAF analyzed with ANOVA and Dunken test: The mean of GAF is significantly different between undifferentiated schizophrenia and paranoid schizophrenia. Moreover it is true about the paranoid type and residual schizophrenia. Although the total quality of life score in paranoid type is higher than others, it is not statistically significant. In addition the analyses revealed that the quality of life and it’s domains did not differ significantly in marital status, age of onset, times of hospitalization, season of birth, history of psychiatric disorders in first degree relevant, and smoking. 

4- There is a moderate correlation between two questionnaires, total score QLS and score GAF with Pearson’s correlation test (p<0.01, r=0.2). 

## Discussion

The result of this study indicated that occupational functioning is significantly different between single and married Schizophrenic patients. The married patients showed the better occupational functioning which could be affecting improvement of their quality of life. Moreover this domain had lower score in residual schizophrenia compared to other types. 

These results coincides with the results of Christen (2005) (21) and Jean (2005) (4) that having well occupational position could be a predictable factor for higher quality of life. And in a way could indicate the correlation between patients’ global functioning and quality of life. 

In our study the educational level was effective on GAF mean in a way that schizophrenic patients with high school or university education had higher scores compared to none-educated patients. 

Jean (2005) (4) also revealed that education level have a positive effect on psychological functioning. Although we need a case-control study to indicate the role of educational level on patient’s prognosis, there was no relation between educational level and quality of life in the study by Bayanzadeh and colleagues and Kalatejari and colleagues (20, 22). 

Consistent with previous research, in our study the mean of GAF is 32.5 which indicated the low Global functioning in most of the patients. For example Christine in his study indicated that 19.8% of schizophrenic participants had good or very good global function (GAF 71-100), 38.2% had bad or partially bad (GAF 41-70) and 42% had very bad global functioning (GAF 0-40). Moreover, in Kraus & Müller-Thomsen’s study (1993) the good result was found only in 23% of participants (23). In Martin’s study (2005) (24), which consisted the fifteen years fallow up of schizophrenic patients, most of the patients had poor prognosis. These results are consistent with the previous findings of some studies such as Shang research (1979) (25, 26) and Mac Glashan (1984) (27). 

In Nabdel study (2002) (28) the GAF scores in most of the patients (about 80%) were less than 40 and more than 90% of patients have had significantly retrograde prognosis. Also, In Barbara’s 21 years fallow up 74% of participants had poor social and economical statuses (29). The results of present study showed that the types of schizophrenia could affect 5 domains which were asked. In a way that paranoid type got the highest average among different types in most of the domains. Although these results were compatible with the reports of some studies (1, 2), but were in contrast with Jean’s study (2005) (4) which could not find any relationship between schizophrenia types and QLS. 

In our study, gender could be effective on quality of life and global functioning. It means the life situation in men is significantly better than women. These results are in contrast with those of other studies (4, 17, 30) that showed higher quality of life among women. Perhaps, the social and cultural problems could be affected on these different results. 

Considering that quality of life is totally poor among schizophrenic patients, patients’ empowerment, teaching the social skills and family supportive care are certain necessities. 

On the other hand moderate regression between quality of life and global functioning, which were not evaluated in any other studies until now, showed the value and reliability of GAF-that is usually used for every psychiatric patient for V axis clinical diagnosis- and indicated the clinical value of this scale. 

## Authors' Contributions

SHH conceived and designed the evaluation and drafted the manuscript. MKY participated in designing the evaluation and interviewed with patients. Both authors read and approved the final manuscript.

## Notes

**Declaration of Interest:** None.

**Clinical Trial Registration: **URL: http://www.Irct.ir/Unique identifier: IRCT201104251457N8
